# Dual Effects of Cigarette Smoke Extract on Proliferation of Endothelial Progenitor Cells and the Protective Effect of 5-aza-2′-deoxycytidine on EPCs against the Damage Caused by CSE

**DOI:** 10.1155/2014/640752

**Published:** 2014-02-18

**Authors:** Zhi-Hui He, Ping Chen, Yan Chen, Ying-Qun Zhu, Sheng-Dong He, Ji-Ru Ye, Da Liu, Yue Yang

**Affiliations:** ^1^Intensive Care Unit, The Second Xiangya Hospital, Central-South University, No. 139 Middle Renmin Road, Changsha, Hunan 410011, China; ^2^Department of Respiratory Medicine, The Second Xiangya Hospital, Central-South University, No. 139 Middle Renmin Road, Changsha, Hunan 410011, China; ^3^Division of Respiratory Disease, Department of Internal Medicine, The Second Xiangya Hospital, Central-South University, No. 139 Middle Renmin Road, Changsha, Hunan 410011, China; ^4^Department of Respiratory Medicine, The Third Hospital of Changsha, No. 176 West Laodong Road, Changsha, Hunan 410015, China; ^5^Department of Geriatric Medicine, The Second Xiangya Hospital, Central-South University, No. 139 Middle Renmin Road, Changsha, Hunan 410011, China

## Abstract

Cigarette smoke is a major public health problem associated with multitude of diseases, including pulmonary and vascular diseases. Endothelial progenitor cells (EPCs) contribute to neovascularization and play an important role in the development of these diseases. The effect of CSE on EPCs is seldom studied. The aim of the current study is to observe the effect of CSE on biological behavior of EPCs and, further, to search for potential candidate agent in protection of proliferation of EPCs against the damage caused by CSE exposure *in vitro*. * Methods*. The proliferations of EPCs isolated from bone marrow of C57BL/6J mice were assessed by MTT after incubating the EPCs with a series of concentrations of CSE (1.0%, 2.5%, 5.0%, and 10.0%) for different times (3, 6, and 24 hours) as well as with 1.0% CSE in presence of 5-AZA-CdR for 24 hours. *Results*. The proliferations of EPCs were significantly enhanced after 3 hours of exposure to concentrations of 1.0% and 2.5% CSE but depressed when exposed to concentrations of 5.0% and 10.0% CSE. Furthermore, the 5-AZA-CdR in concentrations of 2.0 **μ**mol/L and 5.0 **μ**mol/L partly protected against the depression of proliferation of EPCs caused by CSE exposure. *Conclusions*. The CSE showed dual effects on proliferation of EPCs isolated from mice. The 5-AZA-CdR partly protected the proliferation of EPCs against the damage caused by CSE exposure *in vitro*, suggesting that DNA methylation may be involved in the dysfunction of EPCs induced by CSE.

## 1. Introduction

Cigarette smoke (CS) is well known to be a risk factor for pulmonary diseases including chronic obstructive pulmonary disease (COPD), lung cancer, asthma, pulmonary hypertension, and vascular diseases such as atherosclerosis [1]. It is a mixture of more than 4,000 different chemical compounds, such as free radicals, toxins, and electrophiles [2, 3]. The CS extract (CSE) contains almost all of the compounds inhaled by cigarette smokers, including nicotine that is one of the most active pharmacological compounds in CSE.

The bone-marrow-derived endothelial progenitor cells (EPCs) provide an alternative source of endothelial cells (ECs) that contributes to neovessel formation in endothelium structure [4, 5]. The EPCs plays critical role in postnatal vasculogenesis through pivotal bioactivities of mobilization, homing, migration, differentiation, and proliferation in angiovasculogenic tissues [6]. Normal proliferation of EPCs is essential to maintain the efficient count of EPCs in postnatal vasculogenesis [7]. However, the EPCs were greatly reduced in patients with severe COPD and the reduction was correlated with COPD severity [8]. Our previous study also showed decreased and dysfunctional circulating EPCs in patients with COPD [9]. The EPCs seem to be impaired and thus lead to the repair of capacity of the lung tissue reduced in patients with COPD.

Through the studies of evaluating the effects of CSE on various types of cells, such as pulmonary endothelial cell [10], lung fibroblasts [11], epithelial cells [12], airway smooth muscle cells [13] and alveolar macrophages [14], the pathogeneses of COPD relevant to cellular level were gradually elucidated. However, little information is known about the effects of CSE on EPCs concerning the pathophysiology of COPD. The method of stimulating isolated cells with CSE *in vitro* has been explored and frequently applied to determine the direct causes in the relationships between cigarette smoking and cellular functions [15]. In addition, the in-depth study on genome-wide epigenetics gained more comprehensive understandings of epigenome in many diseases. Soria et al. demonstrated that a total of 32% of the bronchial brush samples from former cigarette smokers had methylation [16]. Kikuchi et al. reported that hypermethylations of genes were associated with cigarette smoke [17]. In this study, in attempt to provide fresh information about the impact of CS on proliferation of EPCs to elucidate the pathophysiological mechanisms of the diseases related to CS in cellular level, we assessed the proliferation of EPCs after interfering the cells with a series of concentrations of CSE for various times of exposure *in vitro*. In the meantime, we examined whether a DNA methyltransferase inhibitor, 5-aza-2′-deoxycytidine (5-AZA-CdR), was able to reverse the suppression of EPCs caused by CSE exposure. This study was prospected to provide a new vision into the pathogeneses of pulmonary diseases relevant to smoke in cellular level.

## 2. Methods

### 2.1. Animals

Total of 36 male C57BL/6J mice aged four to six weeks old were randomly enrolled in this study. All animals were purchased from Shanghai laboratory animal center of Chinese Academy of Sciences (Shanghai laboratory animal center of Chinese Academy of Sciences, SLACCAS, Shanghai, China) and fed in a cleaning unit with 23~25 degrees of Celsius (°C), 50~60% humidity, and 12 hours (h) rhythm of night and day. The mice were sacrificed by cervical dislocation. The study was approved by the Institutional Review Board of Central-South University and conformed to the guiding principles for research involving animals and human beings (World Medical Association and American Physiological Society, 2002).

### 2.2. Preparation of CSE

The CSE was prepared according to a previously published method with a minor modification [10]. Briefly, one nonfiltered Fu-Rong cigarette (Tar 13 mg/cigarette, China Tobacco Hunan Industrial Co., Ltd., Changsha, China) was burned and the smoke passed through 20 mL of endothelial growth medium-2 (EGM-2) free of fetal bovine serum (FBS) by connecting to a vacuum pump. This product was supposed to be 100% CSE solution, which was further adjusted with 1 mmol/L NaOH up to 7.4 of pH and filtered through a filter with 0.22 *μ*M pores (Fisher, Hampton, NH, USA) to remove particles and bacteria. This mother CSE solution was diluted with the FBS-free EGM-2 to a series of concentrations (1.0%, 2.5%, 5.0%, and 10.0%) for our following experiments. The preparation of CSE solution was performed freshly for each set of experiments.

### 2.3. Preparation of 5-aza-2′-deoxycytidine (5-AZA-CdR)

Five gram of 5-AZA-CdR powder (Sigma, USA) was dissolved in 2 mL FBS-free EGM-2 solution. This mother liquor was further diluted to a series of concentrations (2.0 *μ*mol/L, 5.0 *μ*mol/L, and 10.0 *μ*mol/L) with FBS-free EGM-2 solution and then stored under minus 80°C until experiments.

### 2.4. Isolation and Culture of EPCs

The ficoll density gradient centrifugation with Histopaque-1083 (Sigma, America) was used to isolate mononuclear cells (MNCs) from bone marrow of C57BL/6J mice according to previously published method [18, 19]. The isolated MNCs were cultured with EGM-2 in presence of 5% FBS (SingleQuots, Lonza, Switzerland) under an atmosphere of 95% humidity and 5% CO_2_ at 37°C practically for culture of EPCs. The cells were inoculated into culture flask with density of (3~5) × 10^6^/mL. Then the culture fluid was replaced totally by fresh culture medium in day 4 of the culture to remove the unattached cells. Then half replacement by the fresh medium was performed every three days. The cell harvest was performed on day 7 of the culture.

### 2.5. Identification of EPCs

**Figure 1:**
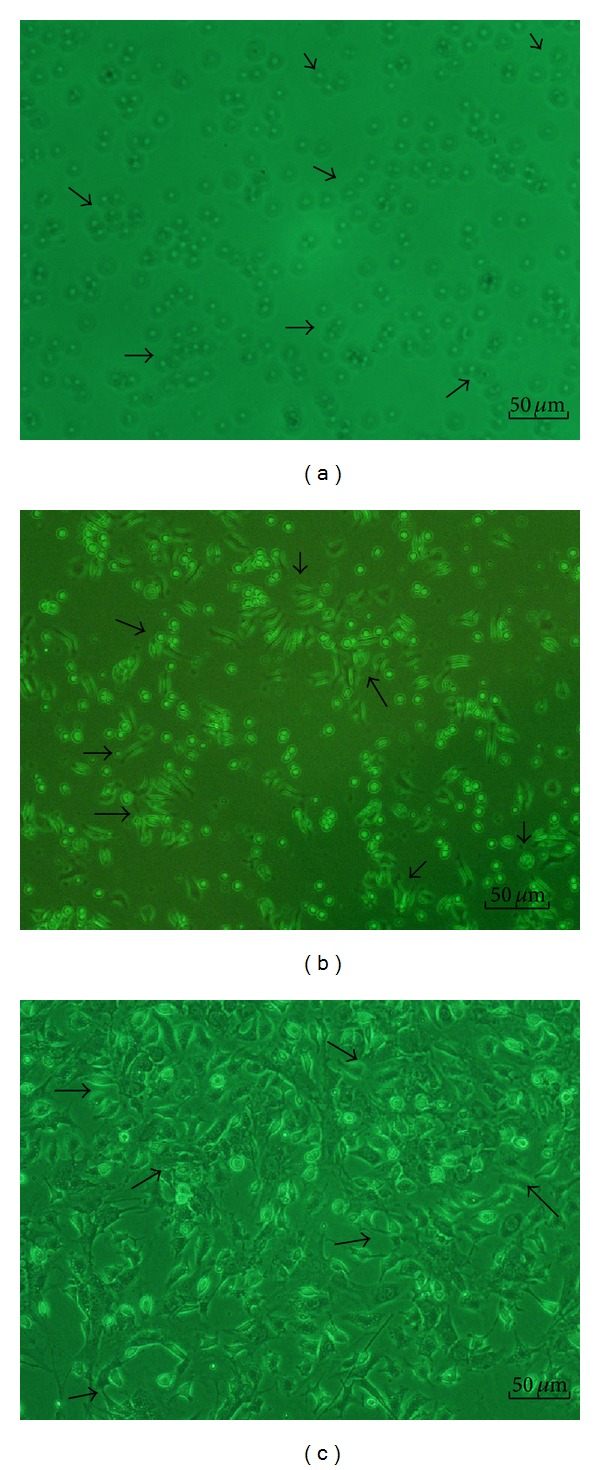
The morphological changes of endothelial progenitor cells (EPCs) during culture. (a) representative photomicrographs of EPCs on day 1 of the culture. The EPCs formed round, the sizes of cells were almost same, and the cells were suspended in the culture medium (arrow 1); (b) on day 4 of the culture, the cells were attached to each other, the sizes were getting enlarged, and the shapes became oval, spindle, or polygonal (arrow 2); (c) on day 7 of the culture, the cells shaped to fusiform or polygon patterns and contacted each other to attempt to form capillary structure (arrow 3). Scale bar represents 50 *μ*m.

**Figure 2:**
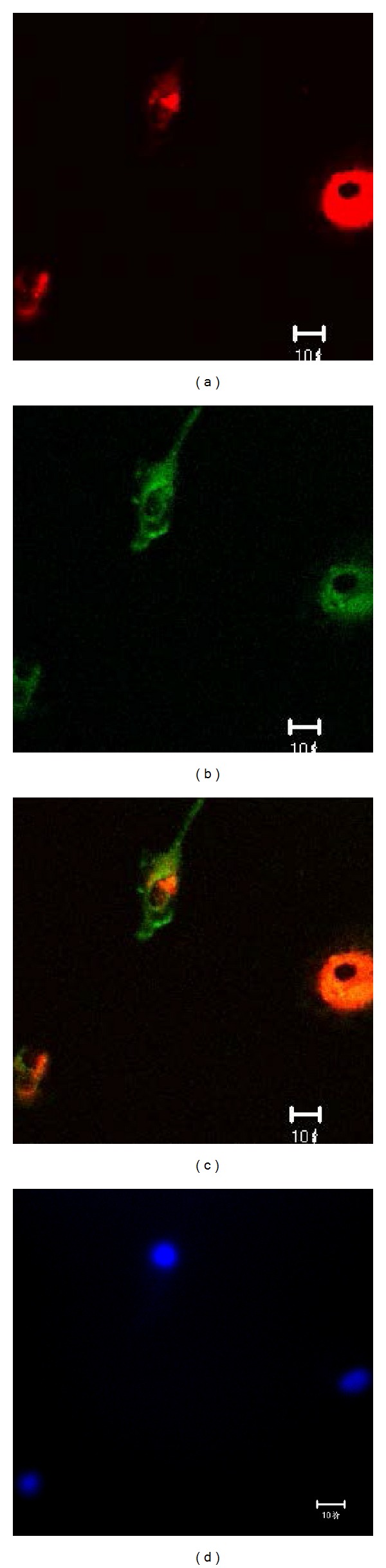
The identification of EPCs by double positive staining with 1,1′-dioctadecyl-3,3,3′,3-tetramethylindocarbocyanine perchlorate (Dil) labeled acetylated low density lipoprotein (Dil-acLDL) and FITC labeled ulex europaeus agglutinin-1 (FITC-UEA-1). The laser scanning confocal microscope (LSCM) illustrated that the cells on day 7 of the culture displayed red cytoplasm when stained with Dil-acLDL (a), green cytomembrane when combined with FITC-UEA-1 (b), orange when double positively stained with Dil-acLDL and FITC-UEA-1) (c), and blue when stained with DAPI in nuclear localization (d). Scale bar represents 10 *μ*m.

We used three methods to identify the EPCs in this study. Firstly, the photos were taken during the culture using phase contrast microscope (Olympus, Japan) to confirm the morphology of EPCs (Figure 1). Secondly, the cells positively stained with both acetylated low density lipoprotein (acLDL) and ulex europaeus agglutinin-1 (UEA-1) were identified as EPCs (Figure 2). The dual staining in cells for 1,1′-dioctadecyl-3,3,3′,3-tetramethylindocarbocyanine perchlorate (Dil)-labeled acLDL (Dil-acLDL) and FITC-labeled UEA-1 (FITC-UEA-1) was performed on day 7 of the culture according to previously described method with a minor modification [20, 21]. The cells were firstly incubated with 7.5 *μ*g/mL Dil-acLDL (Molecular Probes, USA) at 37°C for 4 h and later fixed with 4% paraformaldehyde for 10 minutes (min). After being washed, the cells were treated with 10.0 *μ*g/mL FITC-UEA-1 (Sigma, USA) for 30 min. Finally, the cells were treated with 1 *μ*g/mL 4′,6-diamidino-2-phenylindole (DAPI) for 5 min. The laser scanning confocal microscope (LSCM, Olympus, Japan) was used for the following observation, differentiation, and identification. Fifteen random view-fields were involved to calculate the positive rate of amphophilic cells. Thirdly, the cells concurrently with surface markers of CD34^+^/CD133^+^/Flk-1^+^ were identified as EPCs [19, 22]. The cells (2 × 10^5^/mL) on day 7 of the culture were incubated in dark for 30 min at 4°C and then detected the corresponding surface markers by fluorescence-activated cell sorting (FACS) cytometry using FITC-conjugated anti-mouse CD34 antibody (FITC-CD34, Becton, Dickinson, USA), PE-conjugated anti mouse CD133 antibody (PE-CD133, eBioscience, USA), and APC-conjugated anti-mouse Flk-1 antibody (APC-Flk-1, Becton, Dickinson, USA) according to the instructions. Cells of coexpressing FITC-CD34, PE-CD133, and APC-Flk-1 were identified as EPCs (Figure 5).

### 2.6. Detection of the Proliferation of EPCs Incubated with a Series of Concentrations of CSE for Different Times

The proliferation of EPCs was assessed by 3-(4,5-dimethylthiazol-2-yl)-2,5-diphenyltetrazolium bromide (MTT) (Sigma, USA) assay according to previously described method with a minor modification [23]. The EPCs were trypsinized (Amresco, USA) and centrifuged at 1,500 rpm for 5 min. After being washed, the cells were resuspended with vehicle control. Then the EPCs (1 × 10^4^ in 200 *μ*L volume) were transplanted to 15 wells in each of three 96-well plates. All three plates were incubated under standard cell culture condition at 37°C, 5% CO_2_, and 95% humidity for 48 h. Then culture media were removed and replaced by 200 *μ*L/well of the vehicle control, 1.0% CSE, 2.5% CSE, 5.0% CSE, and 10.0% CSE correspondingly for each of 3 wells in all the three 96-well plates. The three 96-well plates were incubated for 3 h, 6 h, and 24 h, respectively. After incubating for the corresponding periods, each well was added with 20 *μ*L MTT (5 mg/mL) and incubated for another 4 h. Culture media were removed and replaced by 150 *μ*L dimethyl sulfoxide (DMSO) (Sigma, USA). Then the EPCs were shaken for 10 min to dissolve crystal before optical density (OD) measurement at 490 nm for proliferation of EPCs (ELX800, Bio-Tek, USA).

### 2.7. Detection of the Proliferation of EPCs Incubated with CSE in Presence of 5-AZA-CdR

In order to search for any treatment agents in protecting the proliferation of EPCs against depression caused by CSE, a DNA methyltransferase inhibitor, 5-AZA-CdR, was applied to the EPCs exposed to 1.0% CSE for incubation of 24 h and then the proliferation of EPCs was measured by MTT assay. Considering the clinical significance of cigarette smoke in the development of COPD, the exposure concentration of CSE was determined to be a relatively low at 1.0% CSE and the exposure time was relatively long for 24 h in this experiment. In addition, this exposure condition did not deadly depress the proliferation of EPCs according to our previous pilot study.

The EPCs were trypsinized (Amresco, USA) and centrifuged at 1,500 rpm for 5 min. After being washed, the EPCs (1 × 10^4^/mL in 200 *μ*L volume) were transplanted to one 96-well plate. The control and CSE wells were added with 200 *μ*L of vehicle control, respectively, and the 5-AZA-CdR wells were added with 200 *μ*L of 5-AZA-CdR in concentrations of 2.0 *μ*mol/L, 5.0 *μ*mol/L, and 10.0 *μ*mol/L, respectively. The plate was incubated under standard cell culture condition at 37°C, 5% CO_2_, and 95% humidity for 48 h. Then the culture media were removed and replaced by 200 *μ*L of vehicle control in the control wells, of 1.0% CSE in the CSE wells and all 5-AZA-CdR wells. After incubating for another 24 h, each well was added with 20 *μ*L MTT (5 mg/mL) and incubated for another 4 h. Culture media were removed and replaced by 150 *μ*L DMSO. Then the EPCs were shaken for 10 min to dissolve crystal before OD measurement at 490 nm for proliferation of EPCs (ELX800, Bio-Tek, USA).

### 2.8. Statistical Analysis

Analyses were performed using SPSS for Windows 16.0 (SPSS Inc., Chicago, IL, USA). All data were expressed as means ± standard deviation (SD). Analysis of differences among groups was performed using analysis of variance (one-way ANOVA), followed by post hoc analysis as appropriated. Values of *P* < 0.05 were considered statistically significant.

## 3. Results

### 3.1. Identification of EPCs

**Figure 3:**
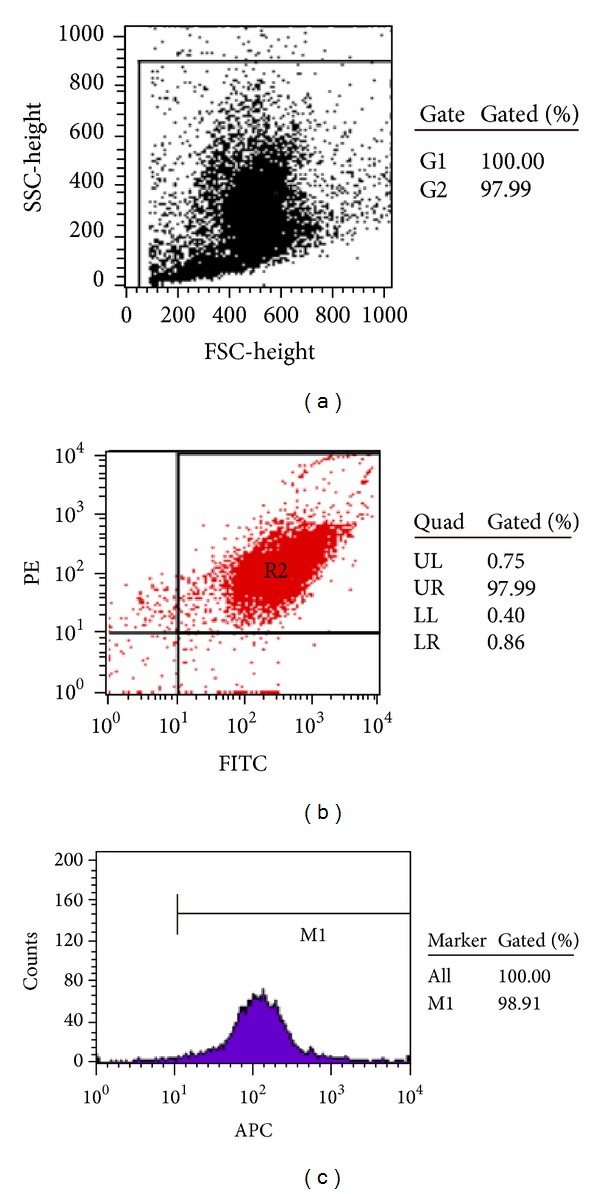
The EPCs coexpressed with surface markers of FITC-CD34^+^, PE-CD133^+^, and APC-Flk-1^+^ detected by flow cytometry. (a) Flow cytometry scatter plot of the test cells. (b) The illustrating example of the gating dot plot used for identification of cells doubly expressed with surface markers of FITC-CD34^+^ and PE-CD133^+^. (c) The positive rate of the surface marker of APC-Flk-1 on the FITC-CD34^+^/PE-CD133^+^-positive cells on day 7 of the culture.

On day 1 of the culture, the MNCs isolated from the murine bone marrow formed round, the sizes of cells were almost same, and the cells were suspended in the culture media (Figure 1(a)). On day 4 of the culture, the cells were attached to each other, the sizes were getting enlarged, and the shapes became oval, spindle, or polygonal. The cells in this stage seemed to tend to gather together to form ball-like structure (Figure 1(b)). On day 7 of the culture, the cells shaped to fusiform or polygon patterns and contacted each other to attempt to form capillary structure (Figure 1(c)). The shapes of cells in this stage were displayed well in the culture media. In addition, the LSCM illustrated that the cells on day 7 of the culture displayed red color in cytoplasm when stained with Dil-acLDL (Figure 2(a)), green color in cytomenbrane when combining with FITC-UEA-1 (Figure 2(b)), and orange color in confocal image when double positively stained with Dil-acLDL and FITC-UEA-1) (Figure 2(c)). The positive rate of amphophilic cells was 94.67 ± 4.16% on day 7 of the culture. Moreover, the rate of cells concurrently expressed with surface markers of FITC-CD34, PE-CD133, and APC-Flk-1 was 95.07 ± 1.73% on day 7 of the culture (Figure 3). Therefore, the cells harvested on day 7 of the culture were believed to be the characteristically biological EPCs and used for the present study.

### 3.2. The Effects of CSE on Proliferation of EPCs

**Figure 4:**
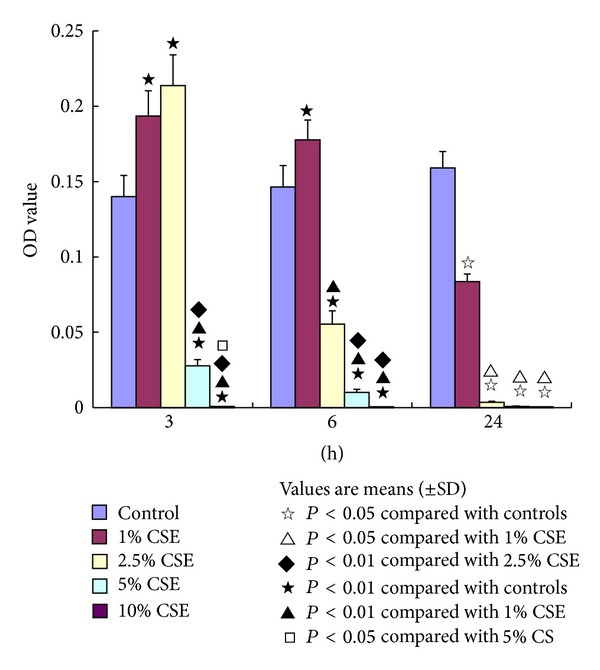
The effects of cigarette smoke extract (CSE) on endothelial progenitor cells (EPCs). The capacity of EPCs (measured by optical density (OD) levels) was significantly increased with stimulations of 1.0% CSE and 2.5% CSE but decreased with interventions of 5.0% CSE and 10.0% CSE for 3 hours (h) compared to that of controls (^★^ *P* < 0.01). The OD levels were significantly increased in EPCs with stimulation of 1.0% CSE but decreased in the cells with interventions of 2.5% CSE, 5.0% CSE, and 10.0% CSE for 6 h compared to those of controls (^★^ *P* < 0.01). The OD levels were significantly decreased in the EPCs with interventions of 1.0% CSE, 2.5% CSE, 5.0% CSE, and 10.0% CSE for 24 h compared to those of controls (^*☆*^ *P* < 0.05).

As shown in Figure 4, the OD levels were significantly increased in the EPCs exposed to both 1.0% CSE (0.1935 ± 0.0168) and 2.5% CSE (0.2136 ± 0.0203) but decreased in the cells exposed to both 5.0% (0.0278 ± 0.0041) and 10.0% CSE (0.0009 ± 0.0001) compared to the OD levels of controls (0.1401 ± 0.0141, *P* < 0.01) for acute exposure to 3 h. In addition, it seems that the higher exposure concentration of the CSE, the lower OD values of the EPCs, showing that the OD levels were significantly lower in the EPCs exposed to 10.0% CSE than the cells exposed to 5.0% CSE (*P* < 0.05), and the later was significantly lower than the cells exposed to 2.5% CSE (*P* < 0.01).

When exposing the EPCs to CSE for 6 h (subacute exposure), the OD levels were significantly increased in the EPCs incubated with 1.0% CSE (0.1777 ± 0.0130) but decreased in the cells incubated with 2.5% CSE (0.0555 ± 0.0087), 5.0% CSE (0.0102 ± 0.0021), and 10.0% CSE (0.0007 ± 0.0002) compared to those of controls (0.1463 ± 0.0143, *P* < 0.01). In addition, the OD values were significantly lower in the EPCs incubated with 5.0% and 10.0% CSE than the cells exposed to 2.5% CSE (*P* < 0.01, Figure 4).

Our results also showed that the OD levels were significantly decreased in the EPCs exposed to 1.0% CSE (0.0837 ± 0.0051), 2.5% CSE (0.0035 ± 0.0007), 5.0% CSE (0.0008 ± 0.0002), and 10.0% CSE (0.0007 ± 0.0001) for 24 h (chronic exposure) compared to those of controls (0.1591 ± 0.0108, *P* < 0.05, Figure 4). For this chronic exposure, the OD values did not show significant differences among the cells exposed to 2.5% CSE, 5.0% CSE, and 10.0% CSE concentrations. However, the OD levels were significantly lower in the EPCs exposed to 2.5~10.0% CSE than the cells exposed to 1.0% CSE (*P* < 0.05).

### 3.3. The Effect of 5-AZA-CdR on the EPCs Exposed to CSE

Considering the clinical significance of cigarette smoke in the development of COPD, we observed the effect of 5-AZA-CdR on the EPCs exposed to a low concentration of CSE (1.0%) for a chronic exposure (24 h). In addition, this exposure condition did not deadly depress the proliferation of EPCs according to the results described above.

**Figure 5:**
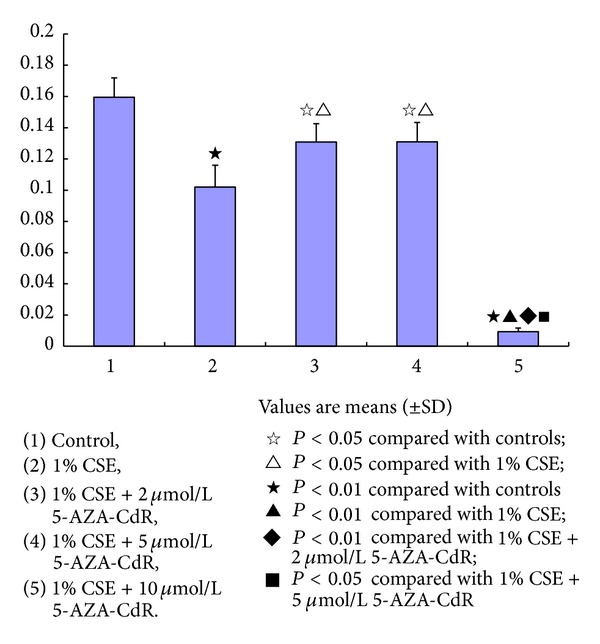
The effect of 5-aza-2′-deoxycytidine (5-AZA-CdR) on the endothelial progenitor cells (EPCs) exposed to cigarette smoke extract (CSE). The OD levels were significantly decreased in all the EPCs in presence of 1.0% CSE, 1.0% CSE + 2 *μ*mol/L 5-AZA-CdR, 1.0% CSE + 5 *μ*mol/L 5-AZA-CdR, and 1.0% CSE + 10 *μ*mol/L 5-AZA-CdR compared to those of controls (^★^ *P* < 0.01; or ^*☆*^ *P* < 0.05). However, the OD levels were significantly higher in the EPCs in presence of 1% CSE + 2 *μ*mol/L~5 *μ*mol/L 5-AZA-CdR than the EPCs in presence of 1.0% CSE (^△^ *P* < 0.05). The high concentration of 5-AZA-CdR (10 *μ*mol/L) showed toxic effect on the EPCs.

As shown in Figure 5, the OD levels were significantly decreased in the EPCs exposed to 1.0% CSE (0.1020 ± 0.0140), 1.0% CSE + 2.0 *μ*mol/L 5-AZA-CdR (0.1308 ± 0.0118), 1.0% CSE + 5.0 *μ*mol/L 5-AZA-CdR (0.1310 ± 0.0124), and 1.0% CSE + 10.0 *μ*mol/L 5-AZA-CdR (0.0093 ± 0.0023) compared to those of the controls (0.1595 ± 0.0125, *P* < 0.05 for all). However, the OD levels were significantly higher in the EPCs exposed to 1.0% CSE + 2.0 *μ*mol/L 5-AZA-CdR and 1.0% CSE + 5.0 *μ*mol/L 5-AZA-CdR than the EPCs exposed to 1.0% CSE (*P* < 0.05), respectively, suggesting that 5-AZA-CdR in concentrations of 2.0 mol/L and 5.0 *μ*mol/L significantly protected against the depression of proliferation of EPCs caused by CSE exposure. There was no significant difference of the OD levels between the EPCs exposed to 1.0% CSE + 2 *μ*mol/L 5-AZA-CdR and the cells exposed to 1.0% CSE + 5.0 *μ*mol/L 5-AZA-CdR. It seems that 5-AZA-CdR in concentration higher than 10.0 *μ*mol/L showed toxic effect on the proliferation of EPCs. Because the OD levels were significantly lower in the EPCs exposed to 1.0% CSE + 10.0 *μ*mol/L 5-AZA-CdR (0.0093 ± 0.0023) than the EPCs exposed to vehicle control of FBS-free EGM-2 (0.1595 ± 0.0125, *P* < 0.05) and 1.0% CSE (0.1020 ± 0.0140, *P* < 0.05), respectively.

## 4. Discussion

The most important finding of this study was that the proliferation capacity of EPCs was significantly enhanced when acute exposing (3 h) the cells to relatively low concentrations of CSE (1.0~2.5%) but depressed when acute exposing the cells to relatively high concentration of CSE (5.0~10.0%) compared to that of EPCs exposed to control medium. These astonishing results suggested that the acute stimulation by a relatively low concentration of CSE might activate certain “danger signals” in the EPCs to motivate the endogenous repair mechanisms of EPCs by which the proliferation of EPCs could be compensated and maintained to struggle against the damages caused by CSE exposure. These protective phenomena can still be observed in the EPCs when they are sub-acutely exposed (6 h) to a relatively low concentration of 1.0% CSE but vanished when chronically exposed (24 h) to any level of CSE, indicating fatal damages in the proliferation of EPCs after a chronic CSE exposure. A DNA methyltransferase inhibitor, 5-AZA-CdR, might partly protect the proliferation of EPCs from damages by chronic CSE exposure.

EPCs were isolated primarily from peripheral blood in 1997 by Asahara et al. [24] and now can also be isolated from bone marrow and umbilical cord blood [25, 26]. EPCs were localized mainly in the bone marrow of postnatal life [27]. In previous studies, EPCs were identified based on their morphology and growth characteristics [28] or characterized by dual staining with Dil-acLDL and FITC-UEA-1 through LSCM observation [21] or defined as three-color fluorescence flow cytometry using antibodies against CD133, CD34, and KDR (Flk-1 in animal) [19]. In present study, EPCs were identified by morphology, dual staining by Dil-acLDL and FITC-UEA-1, and surface markers of CD34^+^/CD133^+^/Flk-1^+^ simultaneously. The current EPC product demonstrated that the highly purified EPCs could be obtained by culturing the isolate MNCs of mice bone marrow with EGM-2 through ficoll density gradient centrifugation.

The endothelium regulates vascular homeostasis and is responsible for angiogenesis in physiological and pathological tissues of humans as well as animals. EPCs are the precursors of endothelial cells and play a fundamental role in the maintenance of endothelial integrity and function by both developing into endothelial cells and secreting vasoactive substances [29, 30]. Accumulating evidences indicated that EPCs derived from bone marrow contributed to reendothelialization of injured vessels as well as neo-vascularization of ischemic lesions in either direct or indirect pathways under physiological or pathological conditions [31, 32]. The proliferation of EPCs is required for tissue repairing and airway remodeling in lungs [33–35]. It was demonstrated that the number and/or function of EPCs were inversely correlated with risk factors of cardiovascular and pulmonary diseases [36, 37]. Cigarette smoke is a major risk factor of cardiovascular and pulmonary diseases. It reduces the number and function of EPCs [38] probably through oxidative stress induced by reactive oxygen species (ROS) from CS [39, 40]. Indeed, the present results demonstrated that a high concentration of CSE harmfully depressed the proliferation of EPCs. Supportably, a study on the proliferation, migration, cytokine release, and contraction of human bronchial smooth muscle cells (SMCs) similarly showed that treatment of the cells with 10.0% CSE induced cell death, reduced migration, and contraction through an increased ROS production [41].

Surprisingly, we found that the proliferation capacity of EPCs was enhanced with stimulation of relatively low concentrations of CSE (1.0~2.5%) for an acute exposure of 3 h. Up to date, the effects of CSE on the cell proliferations were controversially reported. Ambalavanan et al. [42] reported that CSE caused a dose- and time-dependent decreases in neonatal porcine vascular SMCs. Studies also evidenced that CSE induced apoptosis of human airway SMCs [43, 44], human pulmonary endothelial cells [10], and vascular endothelial cells [45]. In controversy, Xing and his colleagues [46] reported that stimulation of rat pulmonary artery SMCs with CSE significantly increased cell proliferation and promoted cell cycle progression. Promotions of cell proliferations stimulated by CSE were also observed on bovine tracheal SMCs [47] and human aortic SMCs [3]. Such controversial results were theoretically explained by discrepancies among the methods applied to those studies, such as the animals, the cell types, and the CSE preparations.

The present finding about the dual effects of CSE on proliferation of EPCs was also supported by clinical observations that the circulating EPCs number was increased and the EPCs were motivated to contribute to vascular repairing and reconstruction of lung vessels in patients with early stage of COPD [48] and that the circulating EPCs number was decreased in patients with later stage of COPD [8, 49].

5-AZA-CdR is a deoxynucleoside analog of cytidine in which the carbon 5 position of the pyrimidine ring is substituted by nitrogen [50]. It is an S-phase specific inhibitor of DNA methyltransferase, which triggers demethylation leading to a consecutive reactivation of epigenetically silenced tumor suppressor genes *in vitro* and *in vivo*. The sparking interest of 5-AZA-CdR is using it as a potential therapeutic agent. In this study, 5-AZA-CdR in concentrations of 2.0 *μ*mol/L and 5.0 *μ*mol/L partly protected the proliferation of EPCs from damage caused by CSE; whereas, 5-AZA-CdR in a high concentration of 10.0 *μ*mol/L showed toxic effect on the proliferation of EPCs. It might be because of a dual mechanism of demethylation in 5-AZA-CdR with reactivation of silenced genes at low doses and cytotoxicity at high doses [51]. Additionally, a novel dose schedule of 5-AZA-CdR for treatment in patients with cancer is suggested due to the myelosuppression induced by high doses [52]. The 5-AZA-CdR protected the proliferation of EPCs from damage caused by 1.0% CSE exposure for 24 h, which could rule out the possibility of the enhancement of proliferation of EPCs resulted from the 1.0% CSE exposure for 3 h observed in this study (Figure 5). In the meantime, this finding suggested that DNA methylation may be involved in the dysfunction of EPCs induced by CSE. Clinically, hypermethylation was detected in cigarette smokers [53–55]. Moreover, the oxidative stress induced by CS destructed the lung tissue in COPD leading to acquired genetic changes including DNA methylation due to inefficient DNA repair mechanism [56].

## 5. Conclusions 

In summary, we demonstrated for the first time that CSE showed dual effects on proliferation of EPCs and a DNA methyltransferase inhibitor, 5-AZA-CdR, partly protected the proliferation of EPCs from damage caused by CSE. These findings would be helpful in understanding of different voices from various studies that focused on roles of EPCs in cigarette smoke-induced diseases.

## References

[1] Banerjee S, Chattopadhyay R, Ghosh A (2008). Cellular and molecular mechanisms of cigarette smoke-induced lung damage and prevention by vitamin C. *Journal of Inflammation*.

[2] Pryor WA, Stone K (1993). Oxidants in cigarette smoke: radicals, hydrogen peroxide, peroxynitrate, and peroxynitrite. *Annals of the New York Academy of Sciences*.

[3] Xu C-B, Lei Y, Chen Q, Pehrson C, Larsson L, Edvinsson L (2010). Cigarette smoke extracts promote vascular smooth muscle cell proliferation and enhances contractile responses in the vasculature and airway. *Basic and Clinical Pharmacology and Toxicology*.

[4] Kopp H-G, Ramos CA, Rafii S (2006). Contribution of endothelial progenitors and proangiogenic hematopoietic cells to vascularization of tumor and ischemic tissue. *Current Opinion in Hematology*.

[5] Lyden D, Hattori K, Dias S (2001). Impaired recruitment of bone-marrow-derived endothelial and hematopoietic precursor cells blocks tumor angiogenesis and growth. *Nature Medicine*.

[6] Hristov M, Erl W, Weber PC (2003). Endothelial progenitor cells: mobilization, differentiation, and homing. *Arteriosclerosis, Thrombosis, and Vascular Biology*.

[7] Zhao X, Huang L, Yin Y, Fang Y, Zhao J, Chen J (2008). Estrogen induces endothelial progenitor cells proliferation and migration by estrogen receptors and PI3K-dependent pathways. *Microvascular Research*.

[8] Huertas A, Testa U, Riccioni R (2010). Bone marrow-derived progenitors are greatly reduced in patients with severe COPD and low-BMI. *Respiratory Physiology and Neurobiology*.

[9] Yang Y, Gan Y, Cao J (2013). Decreased and dysfunctional circulating endothelial progenitor cells in patients with chronic obstructive pulmonary disease. *Chinese Medical Journal*.

[10] Nana-Sinkam SP, Jong DL, Sotto-Santiago S (2007). Prostacyclin prevents pulmonary endothelial cell apoptosis induced by cigarette smoke. *American Journal of Respiratory and Critical Care Medicine*.

[11] Baglole CJ, Sime PJ, Phipps RP (2008). Cigarette smoke-induced expression of heme oxygenase-1 in human lung fibroblasts is regulated by intracellular glutathione. *American Journal of Physiology*.

[12] Lau WKW, Chan SCH, Law ACK, Ip MSM, Mak JCW (2012). The role of MAPK and Nrf2 pathways in ketanserin-elicited attenuation of cigarette smoke-induced induced iL-8 production in human bronchial epithelial cells. *Toxicological Sciences*.

[13] Smelter DF, Sathish V, Thompson MA, Pabelick CM, Vassallo R, Prakash YS (2010). Thymic stromal lymphopoietin in cigarette smoke-exposed human airway smooth muscle. *Journal of Immunology*.

[14] Pires KM, Lanzetti M, Rueff-Barroso CR (2012). Oxidative damage in alveolar macrophages exposed to cigarette smoke extract and participation of nitric oxide in redox balance. *Toxicology in Vitro*.

[15] Shapiro SD (2004). Smoke gets in your cells. *American Journal of Respiratory Cell and Molecular Biology*.

[16] Soria J-C, Rodriguez M, Liu DD, Jack Lee J, Ki Hong W, Mao L (2002). Aberrant promoter methylation of multiple genes in bronchial brush samples from former cigarette smokers. *Cancer Research*.

[17] Kikuchi S, Yamada D, Fukami T (2006). Hypermethylation of the TSLC1/IGSF4 promoter is associated with tobacco smoking and a poor prognosis in primary nonsmall cell lung carcinoma. *Cancer*.

[18] Purhonen S, Palm J, Rossi D (2008). Bone marrow-derived circulating endothelial precursors do not contribute to vascular endothelium and are not needed for tumor growth. *Proceedings of the National Academy of Sciences of the United States of America*.

[19] Rafat N, Hanusch C, Brinkkoetter PT (2007). Increased circulating endothelial progenitor cells in septic patients: correlation with survival. *Critical Care Medicine*.

[20] Bartsch T, Brehm M, Zeus T, Kögler G, Wernet P, Strauer BE (2007). Transplantation of autologous mononuclear bone marrow stem cells in patients with peripheral arterial disease (The TAM-PAD Study). *Clinical Research in Cardiology*.

[21] Chen JZ, Zhu JH, Wang XX (2004). Effects of homocysteine on number and activity of endothelial progenitor cells from peripheral blood. *Journal of Molecular and Cellular Cardiology*.

[22] Hirschi KK, Ingram DA, Yoder MC (2008). Assessing identity, phenotype, and fate of endothelial progenitor cells. *Arteriosclerosis, Thrombosis, and Vascular Biology*.

[23] Asahara T, Kawamoto A (2004). Endothelial progenitor cells for postnatal vasculogenesis. *American Journal of Physiology*.

[24] Asahara T, Murohara T, Sullivan A (1997). Isolation of putative progenitor endothelial cells for angiogenesis. *Science*.

[25] Chen JK, Deng YP, Jiang GJ, Liu YZ, Zhao T, Shen FM (2013). Establishment of tube formation assay of bone marrow-derived endothelial progenitor cells. *CNS Neuroscience & Therapeutics*.

[26] Murohara T (2010). Cord blood-derived early outgrowth endothelial progenitor cells. *Microvascular Research*.

[27] Shi Q, Rafii S, Wu Hong-De M (1998). Evidence for circulating bone marrow-derived endothelial cells. *Blood*.

[28] Bayat H, Fathi F, Peyrovi H, Mowla SJ (2013). Evaluating the expression of self-renewal genes in human endothelial progenitor cells. *Cell*.

[29] Krenning G, van Luyn MJA, Harmsen MC (2009). Endothelial progenitor cell-based neovascularization: implications for therapy. *Trends in Molecular Medicine*.

[30] Yang Z, von Ballmoos MW, Faessler D (2010). Paracrine factors secreted by endothelial progenitor cells prevent oxidative stress-induced apoptosis of mature endothelial cells. *Atherosclerosis*.

[31] Devanesan AJ, Laughlan KA, Girn HRS, Homer-Vanniasinkam S (2009). Endothelial progenitor cells as a therapeutic option in peripheral arterial disease. *European Journal of Vascular and Endovascular Surgery*.

[32] Zampetaki A, Kirton JP, Xu Q (2008). Vascular repair by endothelial progenitor cells. *Cardiovascular Research*.

[33] Critser PJ, Yoder MC (2010). Endothelial colony-forming cell role in neoangiogenesis and tissue repair. *Current Opinion in Organ Transplantation*.

[34] Loebinger MR, Aguilar S, Janes SM (2008). Therapeutic potential of stem cells in lung disease: progress and pitfalls. *Clinical Science*.

[35] Santos S, Peinado VI, Ramírez J (2002). Characterization of pulmonary vascular remodelling in smokers and patients with mild COPD. *European Respiratory Journal*.

[36] Hill JM, Zalos G, Halcox JPJ (2003). Circulating endothelial progenitor cells, vascular function, and cardiovascular risk. *The New England Journal of Medicine*.

[37] Kondo T, Hayashi M, Takeshita K (2004). Smoking cessation rapidly increases circulating progenitor cells in peripheral blood in chronic smokers. *Arteriosclerosis, Thrombosis, and Vascular Biology*.

[38] di Stefano R, Barsotti MC, Felice F (2010). Smoking and endothelial progenitor cells: a revision of literature. *Current Pharmaceutical Design*.

[39] Jeong Y-Y, Park H-J, Cho Y-W (2012). Aged red garlic extract reduces cigarette smoke extract-induced cell death in human bronchial smooth muscle cells by increasing intracellular glutathione levels. *Phytotherapy Research*.

[40] Yamaguchi Y, Nasu F, Harada A, Kunitomo M (2007). Oxidants in the gas phase of cigarette smoke pass through the lung alveolar wall and raise systemic oxidative stress. *Journal of Pharmacological Sciences*.

[41] Yoon CH, Park H-J, Cho Y-W (2011). Cigarette smoke extract-induced reduction in migration and contraction in normal human bronchial smooth muscle cells. *Korean Journal of Physiology and Pharmacology*.

[42] Ambalavanan N, Carlo WF, Bulger A, Shi J, Philips JB (2001). Effect of cigarette smoke extract on neonatal porcine vascular smooth muscle cells. *Toxicology and Applied Pharmacology*.

[43] Hu W, Xie J, Zhao J, Xu Y, Yang S, Ni W (2009). Involvement of Bcl-2 family in apoptosis and signal pathways induced by cigarette smoke extract in the human airway smooth muscle cells. *DNA and Cell Biology*.

[44] Oltmanns U, Chung KF, Walters M, John M, Mitchell JA (2005). Cigarette smoke induces IL-8, but inhibits eotaxin and RANTES release from airway smooth muscle. *Respiratory Research*.

[45] Chen Y, Luo H, Kang N (2012). Beraprost sodium attenuates cigarette smoke extract-induced apoptosis in vascular endothelial cells. *Molecular Biology Reports*.

[46] Xing AP, Du YC, Hu XY (2012). Cigarette smoke extract stimulates rat pulmonary artery smooth muscle cell proliferation via PKC-PDGFB signaling. *Journal of Biomedicine and Biotechnology*.

[47] Pera T, Gosens R, Lesterhuis AH (2010). Cigarette smoke and lipopolysaccharide induce a proliferative airway smooth muscle phenotype. *Respiratory Research*.

[48] Peinado VI, Ramírez J, Roca J, Rodriguez-Roisin R, Barberà JA (2006). Identification of vascular progenitor cells in pulmonary arteries of patients with chronic obstructive pulmonary disease. *American Journal of Respiratory Cell and Molecular Biology*.

[49] Palange P, Testa U, Huertas A (2006). Circulating haemopoietic and endothelial progenitor cells are decreased in COPD. *European Respiratory Journal*.

[50] Yang X, Lay F, Han H, Jones PA (2010). Targeting DNA methylation for epigenetic therapy. *Trends in Pharmacological Sciences*.

[51] Jabbour E, Issa J-P, Garcia-Manero G, Kantarjian H (2008). Evolution of decitabine development: accomplishments, ongoing investigations, and future strategies. *Cancer*.

[52] Karahoca M, Momparler RL (2013). Pharmacokinetic and pharmacodynamic analysis of 5-aza-2′-deoxycytidine (decitabine) in the design of its dose-schedule for cancer therapy. *Clinical Epigenetics*.

[53] Baryshnikova E, Destro A, Infante MV (2008). Molecular alterations in spontaneous sputum of cancer-free heavy smokers: results from a large screening program. *Clinical Cancer Research*.

[54] Kerr KM, Galler JS, Hagen JA, Laird PW, Laird-Offringa IA (2007). The role of DNA methylation in the development and progression of lung adenocarcinoma. *Disease Markers*.

[55] Yanagawa N, Tamura G, Oizumi H, Endoh M, Sadahiro M, Motoyama T (2011). Inverse correlation between EGFR mutation and FHIT, RASSFlA and RUNX3 methylation in lung adenocarcinoma: relation with smoking status. *Anticancer Research*.

[56] Tzortzaki EG, Papi A, Neofytou E, Soulitzis N, Siafakas NM (2013). Immune and genetic echanisms in COPD: possible targets for therapeutic interventions. *Current Drug Targets*.

